# Impact of treated wash water from ready mix concrete plants on concrete properties and durability

**DOI:** 10.1038/s41598-026-39590-5

**Published:** 2026-03-07

**Authors:** Ayman Shamseldein, Mohamed Amr, Fatma Attia

**Affiliations:** 1 Structural Engineering Department, Faculty of Engineering, Ain Shams University, Cairo, Egypt; 2 Civil Engineering Programme, School of Engineering and Computer Science, University of Hertfordshire Hosted by GAF, Cairo, Egypt

**Keywords:** Ready-mix concrete wash water, Wastewater reuse, Mechanical properties, Compressive strength, Concrete electrical resistivity, Durability, Sustainable concrete production, Engineering, Environmental sciences, Materials science

## Abstract

Concrete production consumes nearly 16% of global freshwater resources, highlighting the urgent need for sustainable alternatives to potable water. This study investigates the feasibility of using treated wash water from ready-mix concrete plants as a partial or full replacement for mixing water. Concrete mixes were prepared with 25%, 50%, 75%, and 100% wash water replacement ratios to evaluate the impacts on fresh and hardened properties as well as durability. Electrical resistivity testing was integrated into the experimental programme to assess the durability performance of concrete containing varying wash water contents. The results showed a reduction in workability of up to 50% compared to the control mix prepared with potable water. At 28 days, compressive strength decreased by 15.9%, 17.3%, and 18.3% for mixes containing 25%, 75%, and 100% wash water replacement, respectively. Electrical resistivity increased significantly with higher wash water replacement by 44% and 60% for mixes with 25% and 50% wash water replacement, and by up to six times at full replacement, indicating enhanced durability and resistance to corrosion. Furthermore, empirical equations were developed and validated against the experimental data to estimate the reduction in compressive strength of concrete mixes incorporating treated water. These findings provide performance-based guidance for the broader adoption of treated wash water as a sustainable alternative in concrete production while maintaining acceptable structural and durability performance.

## Introduction

Concrete is a fundamental material of modern infrastructure and one of the most widely used construction materials worldwide^1,2^. The large-scale production of concrete in ready-mix plants requires substantial natural resources, particularly fresh water, and generates significant quantities of wastewater during the cleaning of mixing trucks and batching equipment. This wash water has a high pH value and typically contains cement particles, aggregates, and chemical admixtures, which cause both environmental and economic challenges. Conventional treatment and disposal methods for washing water are costly, while improper discharge can cause soil and water pollution^3^.

Producing one cubic meter of concrete requires about 70 L of wash water for washing operations^4^. With an estimated annual worldwide concrete consumption of 30 billion tons, this translates to nearly 875 billion liters of wash water produced each year. Recycling wash water in concrete production not only reduces the demand for potable water but also creates broader sustainability benefits. The freshwater saved could be redirected to agriculture, with the potential to irrigate up to 218,750 feddans and support more than three million people when advanced irrigation methods are applied^5^. This practice provides two key benefits: reducing the consumption of freshwater resources^6^ and advancing global sustainability goals, notably SDG 6 (Clean Water and Sanitation), SDG 2 (Zero Hunger), SDG 12 (Responsible Consumption and Production), and SDG 13 (Climate Action).

Several studies have investigated the feasibility of recycling wash water from ready-mix concrete plants. Tsimas and Zerkavi^7^ studied the properties of wash water collected from ready-mix concrete plants. They found that all collected water samples had a pH above 11.5, classifying it as hazardous waste unsuitable for direct environmental disposal. However, the same samples met the chemical properties requirements of ASTM C1602^8^ and BS EN 1008^9^ indicating its suitability as mixing water in concrete. Klus et al.^6^ reported that replacing 20% and 50% of mixing water with wash water in concrete production is feasible without negatively impacting the mechanical properties. They observed a reduction in setting time of about 15 min and a slight increase in both flexural and compressive strength at 28 days. Xuan et al.^10^ highlighted that while wastewater composition is generally similar (cement hydration products and fine sand), the solid content varies significantly. They noted that the fine particles can provide a filler effect that enhances durability. They concluded that further studies are required to evaluate the effect of wastewater on concrete durability performance.

Other studies have drawn attention to the impact of using untreated wash water. Ghrair et al.^4^, reported that raw wash water failed to meet the maximum concentration limits required by ASTM C1602^8^, or BS EN 1008^9^ standards and significantly reduced slump values and compressive strength, even after dilution. However, when treated wash water was used, no adverse effects on strength or workability were reported. Al-Joulani et al.^11^ similarly found that replacing 30–40% of fresh water with treated wash water did not significantly affect the concrete strength.

Recent investigations have focused on self-compacting concrete incorporating wash water. Bahraman et al.^12^ investigated the viability of utilising wash water from ready-mix plants and synthetic wastewater with varying total dissolved solids (TDS) levels in the production of self-compacting concrete. The findings indicated that the incorporation of wash water or synthetic wastewater into the concrete mixes reduced the workability. Moreover, the 28-day compressive strength results of all specimens utilising wash water or synthetic wastewater were decreased, except for the mixture with synthetic wastewater of 1000 mg/L TDS, which showed an increase of 13.35%. Utilising the wash water in the self-compacted concrete reduced the compressive strength by 2.87% relative to the control sample. The 28-day flexural strength of the specimens utilising wash water or synthetic wastewater improved, except for synthetic wastewater with TDS levels of 50 and 10,000 mg/L. Moreover, the results of the Scanning Electron Microscope indicated that both wash water and synthetic wastewater with total dissolved solids (TDS) levels of 3000, 5000, and 10,000 mg/L decreased durability and compressive strength. Aruntaş et al.^13^ examined the fresh and mechanical characteristics of concretes produced using wash water from a ready-mix concrete plant. Wash water was utilised at rates of 25%, 50%, 75%, and 100% as a replacement for potable water. The results showed that the workability of specimens mixed with wash water was enhanced in mixes when using superplasticisers at maximume value of 2%. However, the 28-day compressive strength of the mixtures containing 100% wash water decreased by 10% relative to the control mix. They concluded that the utilisation of wash water in concrete production will significantly reduce environmental pollution.

Recently, Maddikeari et al.^14^ conducted a comprehensive review of studies investigating the feasibility of utilizing wastewater resources, including wash water from ready-mix concrete plants, as a sustainable alternative to potable water in concrete production. Their review reported that the use of wastewater generally leads to a reduction in workability and slump values. Compressive strength was found to be negatively affected at early ages; however, strength enhancement can be achieved when the proportioning between potable water and wastewater is properly optimized. Furthermore, the authors emphasized that pre-treatment of wastewater can significantly reduce impurities and consequently improve the mechanical and durability performance of the produced concrete. The review concluded that this research area still requires further investigation, particularly with respect to long-term performance and durability. In addition, the study highlighted the need for developing effective pre-treatment strategies and establishing clear guidelines and standards to ensure the safe and reliable use of treated wastewater in concrete production.

Although several studies have investigated the reuse of wash water from ready-mix concrete plants, results remain inconsistent, particularly regarding its effects on workability and mechanical properties. Very few studies have examined the full replacement of potable water with wash water in concrete production. Moreover, most research has focused on mechanical properties, with limited attention to durability. These gaps highlight the need for a comprehensive study that evaluates not only the fresh and hardened properties of concrete with wash water but also its durability, providing guidance for practical application in sustainable concrete production.

In this study, an experimental program was conducted to evaluate the effects of replacing potable water with wash water collected from a ready-mix concrete plant. Replacement ratios of 25%, 50%, 75%, and 100% were investigated to assess both partial and full replacement. The influence of wash water replacement on key concrete properties, including workability, mechanical performance, and durability, was examined. Electrical resistivity testing was incorporated as a rapid, non-destructive, and portable method to evaluate the durability and corrosion resistance of concrete incorporating wash water. Furthermore, empirical equations were developed and validated to estimate the reduction in compressive strength, providing performance-based guidance for the sustainable use of wash water in concrete production.

The remainder of this paper is structured as follows: Sect. "Experimental programme" describes the experimental programme, including material properties, specimen preparation, and testing procedures. Section "Materials" presents and discusses the results, while Sect. "Wash water treatment" summarizes the main conclusions and implications of the study.

## Experimental programme

An experimental programme was developed to evaluate the influence of wash water from ready-mix concrete plants on concrete properties. Five concrete mixes were prepared: one control mix with 100% potable water and four mixes in which potable water was partially replaced with wash water at ratios of 25%, 50%, 75%, and 100%. These wash water replacement ratios were selected to allow a systematic assessment of both partial and full substitution of potable water. Similar replacement ratios have been adopted in previous studies investigating the reuse of wash water in concrete production^13^, allowing comparison of results and identification of threshold effects on fresh, mechanical, and durability properties of concrete.

Before use, the collected wash water was subjected to chemical analysis to assess its suitability. The prepared concrete mixes were then tested for workability (slump test), mechanical performance (compressive and tensile strength tests), and durability (electrical resistivity test). All concrete specimens were mixed, cast, cured, and tested under the same laboratory conditions, following the same procedures and standards to ensure consistency and comparability across all mixes.

### Materials

In this study, ordinary Portland cement (CEM I 42.5 N), locally produced in Egypt and conforming to BS EN 197–1^15^, was used. Crushed limestone coarse aggregate with a nominal maximum size of 20 mm and siliceous natural sand as fine aggregate were utilised. All concrete mixes were prepared using a tilting drum mixer. The mixture proportions were kept constant for all mixes, with the only variable being the type of mixing water. No additional chemical admixtures or superplasticizers were added to the concrete mixes in order to assess the direct effect of wash water replacement. The only admixture present was that originally contained in the collected wash water. The concrete mix designs followed the British Standards method, and the mixture details are summarized in Table 1.

**Table 1:** Concrete mix proportions.

Concrete sample	Wash water replacement ratio %	Water-cement ratio (W/C)	Cement (Kg/m^3^)	Fine aggregate (Kg/m^3^)	Coarse aggregate (Kg/m^3^)	Water content (L/m^3^)
C	0	0.51	350	850	1100	180
M25	25	0.51	350	850	1100	180
M50	50	0.51	350	850	1100	180
M75	75	0.51	350	850	1100	180
M100	100	0.51	350	850	1100	180

Concrete samples were designed as follows: the control mix (C) used 100% potable water, while other mixes were labeled as “M” followed by the percentage of wash water replacement. For example, M75 denotes a mix in which 75% of the mixing water was replaced with wash water and the remaining 25% was potable water. The wash water was collected from a ready-mix concrete truck in the New Administrative Capital in Cairo, Egypt.

The Concrete mix proportions per cubic meter for the mix from which the wash water was collected are as follows: cement 450 kg/m^3^, sand 650 kg/m^3^, coarse aggregate 1150 kg/m^3^and admixture 6.75 L/m^3^. The used admixture was commercially available and it was retarding admixtures and high-range water reducers (X-Calibur X500), which conform to type G of ASTM C494 standards^16^.

### Wash water treatment

**Fig. 1:**
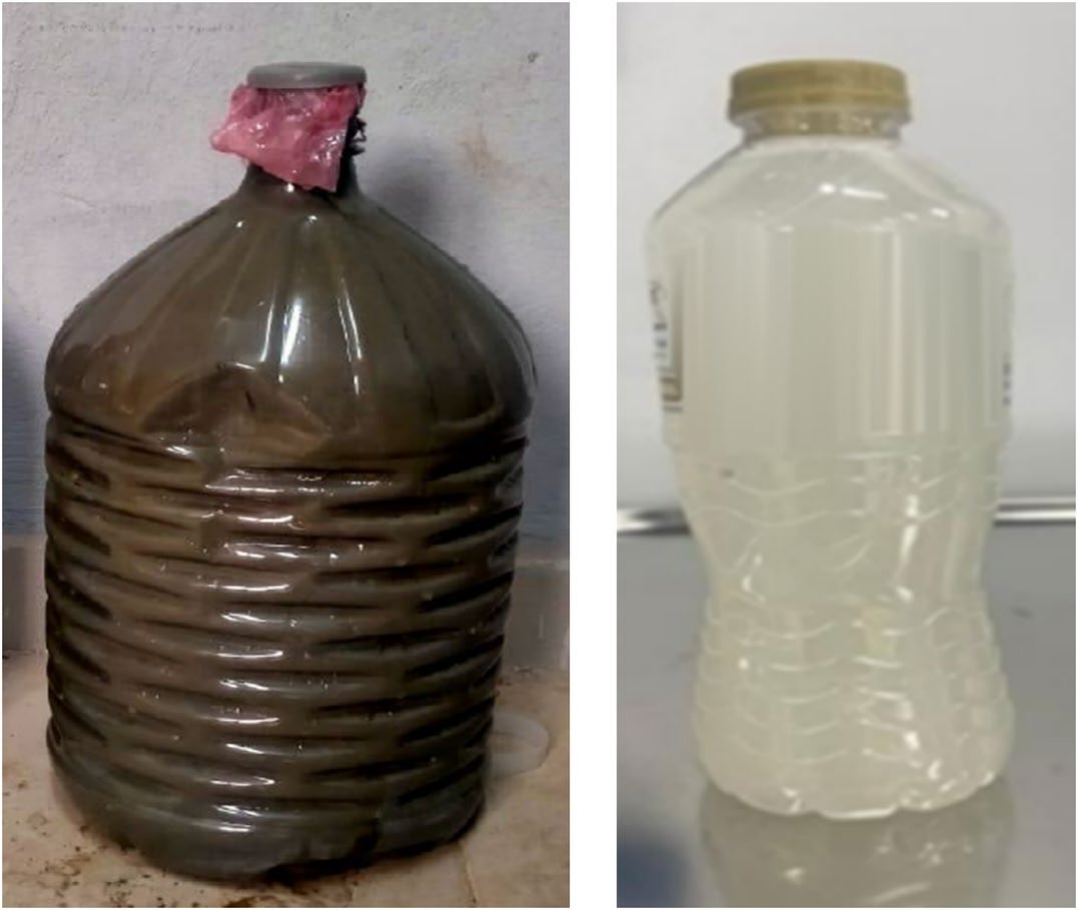
Wash water before and after treatment.

The wash water collected from the concrete transport truck was initially stored in containers. During storage, hardening occurred due to ongoing cement hydration. To mitigate this, a simple treatment procedure was implemented. The wash water was passed through a standard sieve with an opening size of 0.075 mm, which retained larger cementitious particles while allowing finer particles and dissolved solids to pass through. Following filtration, the appearance of the wash water changed from dark brown, indicating a high concentration of suspended solids, to a lighter yellow, reflecting the reduction of the suspended solids. Figure 1 presents the wash water before and after treatment.

### Specimen preparation

The Concrete mixes were prepared using a laboratory-grade tilting drum mixer with a capacity of 0.15 m^3^ (approximately 1/7 cubic meter). Each batch was mixed for 2 min to ensure uniformity. For every mix, six cubes of dimensions 150 × 150 × 150 mm and three cylinders measuring 150 mm in diameter and 300 mm in height were cast. All specimens were prepared in accordance with the requirements of BS EN 12,390^17^.

### Experimental tests

In this study, a series of tests was conducted to assess the impact of using wash water on the properties of concrete. A chemical analysis of the wash water was conducted to characterize its composition and assess its suitability for producing concrete mixtures. The fresh properties of concrete were evaluated using slump tests, while the hardened properties were examined through compressive strength and splitting tensile strength tests. Additionally, long-term performance and durability were evaluated using an electrical resistivity test.

A chemical analysis of the wash water was performed in accordance with ASTM D512^18^ and ASTM D516^19^. Slump tests were performed immediately after mixing according to BS-EN 12,350–2^20^. Compressive strength tests were carried out on cube specimens after curing in potable water for 7 and 28 days, following the procedures of BS EN 12,390^17^. Splitting tensile tests were conducted on cylinder specimens after 28 days of curing, also in accordance with BS EN 12,390^17^.

An electrical resistivity test was conducted in this study to evaluate the durability of concrete mixes produced using wash water. The electrical resistivity test provides a rapid, non-destructive means of assessing the likelihood of corrosion in reinforced concrete structures, as well as the overall quality and homogeneity of the concrete. It also reflects factors such as pore structure, moisture content, and the effectiveness of the curing process.

In this test, an electrical current (I) was applied to the surface of concrete specimens, and the potential difference (ΔV) was measured at specific points using copper electrodes.

**Fig. 2:**
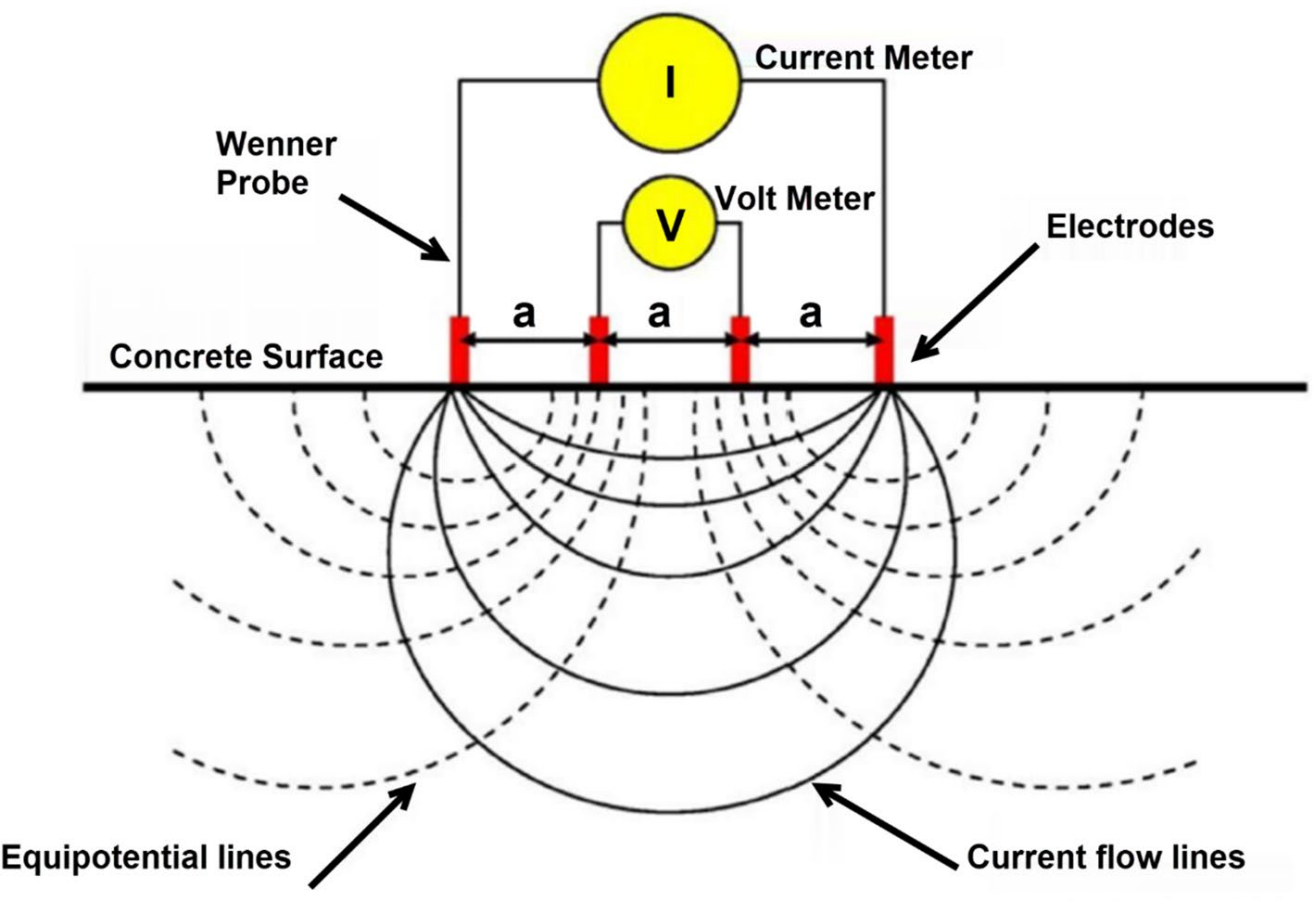
Schematic diagram for the electrical resistivity test.

Figure 2 shows a schematic diagram for the electrical resistivity test. The resistivity of concrete (*ρ*) was then calculated using Eq. (1):

1 $$\rho =2\pi a\frac{\Delta V}{I}$$

where: *ρ* is the electrical resistivity of concrete in KΩ.cm. *I* is the applied electrical current on a flat concrete surface. ΔV is the potential difference measured at specific points on the surface. a is the distance between the applied electrodes.

**Fig. 3:**
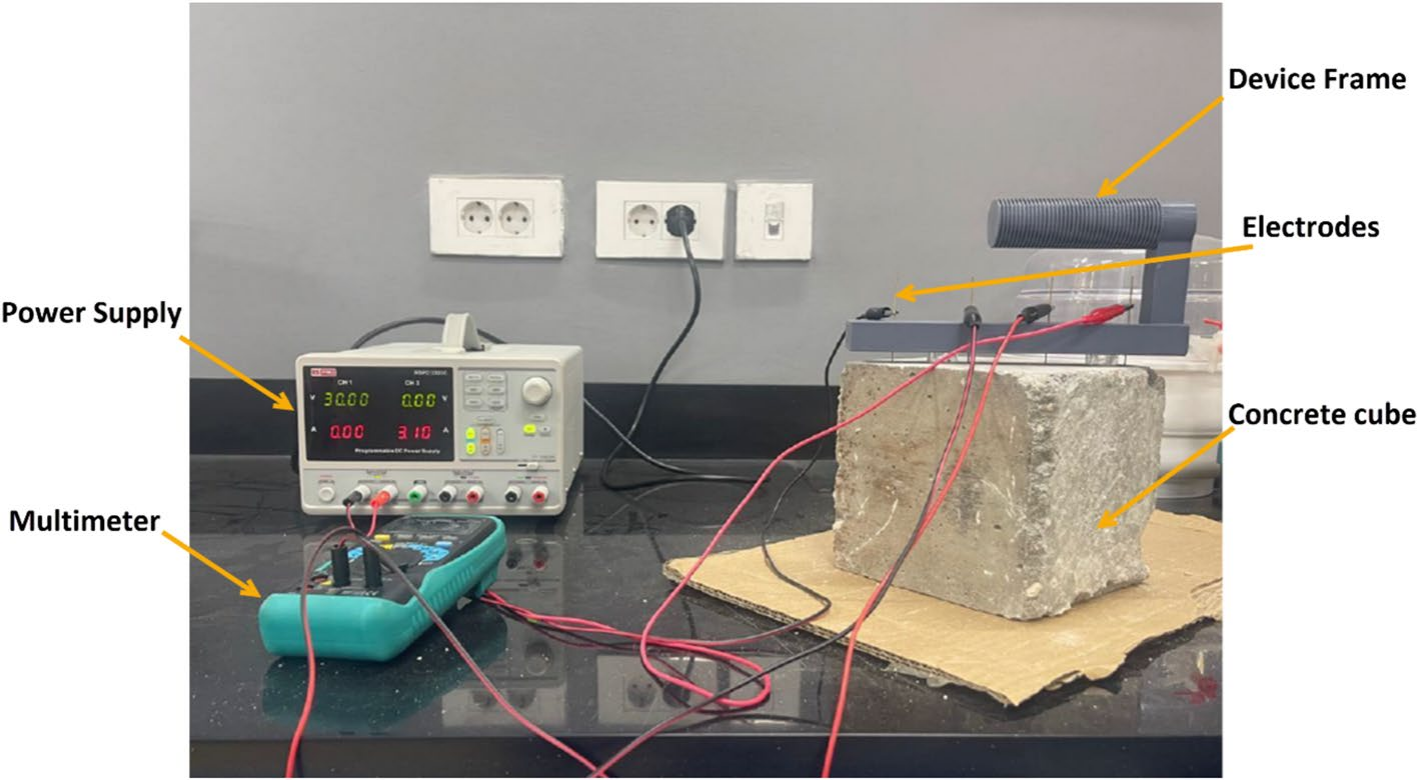
Electrical resistivity test setup.

The electrical resistivity test was conducted on cube specimens under both dry and fully saturated conditions at 28 days of curing. The resistivity measurement device frame was fabricated using additive manufacturing (3D printing). Copper rods with a diameter of 1 mm were embedded into pre-formed holes in the device frame. The experiment was conducted using a direct current (DC) power supply at 30 V, with multimeters used to measure the current and potential difference across the copper electrodes. Figure 3 shows the fabricated electrical resistivity test setup.

## Test results and discussion

### Chemical analysis test

A chemical analysis test was conducted on the wash water collected from the ready-mix concrete plant to assess its potential impact on the properties of fresh and hardened concrete. The results presented in Table 2, were compared with the requirements of BS EN 1008^9^, ASTM C1602^5^ and ECP 203^21^. It was found that all measured values complied with the ECP 203 limits. However, it should be noted that ECP 203 specifications are generally stricter than those of other standards, such as ASTM C1602^8^ and BS EN1008^9^ as also shown in Table 2.

**Table 2:** The chemical analysis of the used wash water.

Parameter	Wash water	ASTM C1602	BS-EN 1008	ECP 203
pH^(1)^	9.1	–	–	> 7
Cl^-1^(mg/l)^(2)^	0.0888	≤ 1.0	≤ 1.0	≤ 0.5
SO_3_^–2^(mg/l)^(3)^	0.30	≤ 2.5	≤ 1.6	≤ 0.3
TDS (mg/l)^(4)^	0.66	≤ 50	–	≤ 2.0

^(1)^pH is the potential of hydrogen

^(2)^Cl^-1^ is chlorine (mg/l)

^(3)^SO_3_^–2^ is sulphate (mg/l)

^(4)^TDS is total dissolved solids (mg/l)

The chemical analysis showed that the treated wash water contained residual solids and exhibited a higher pH than conventional potable water. It is also important to note that when wash water is blended with potable water, the concentration of dissolved salts is expected to decrease, potentially mitigating adverse effects.

### Slump test

**Fig. 4:**
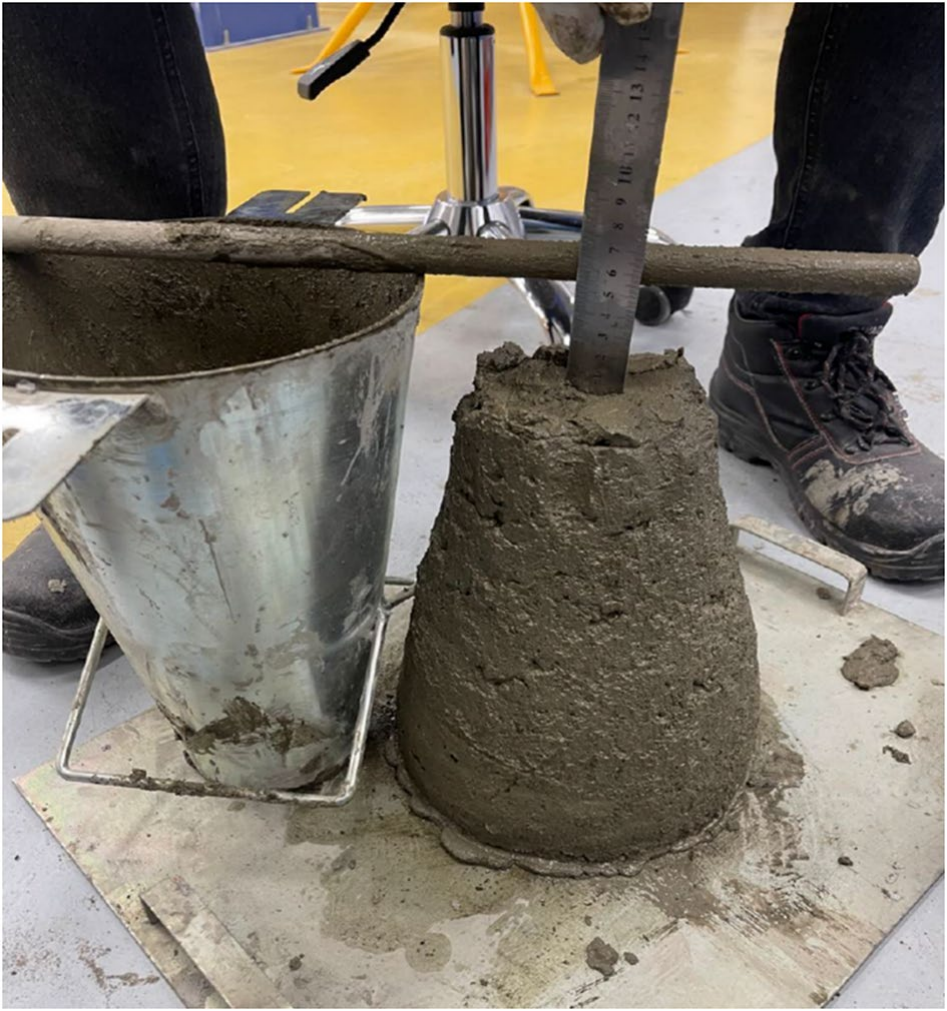
Slump test results for the mix with 25% wash water replacement.

The slump test results indicated a significant reduction in workability when wash water was used in the mixes. While the control mix (prepared with potable water) showed a slump value of 12 cm, all mixes incorporating wash water recorded a slump of 6 cm. Figure 4 presents the slump test results for the mix with 25% wash water replacement ratio. The reduction in workability observed in concrete mixes incorporating wash water can be attributed to the presence of fine suspended cementitious particles and dissolved solids, which increase the overall surface area within the mix and consequently raise the water demand. These fine particles act as micro-fillers that absorb free water and reduce lubrication between aggregates, leading to lower slump values. As a result, the mixes prepared with wash water exhibited noticeably lower workability compared to the control mix.

### Compressive strength test

**Fig. 5:**
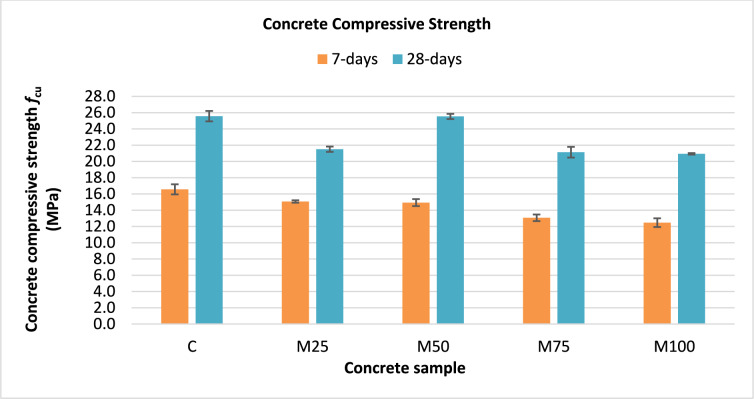
Compressive strength of concrete mixes at 7 and 28 days.

The compressive strength test was carried out on cube specimens at ages of 7 and 28 days for all mixes in accordance with BS EN 12,390^17^. The test results are summarized in Table 3. It can be observed that incorporating wash water into the concrete mixes generally led to a reduction in compressive strength compared to the control mix. At 7 days, mixes with 25% and 50% wash water replacement showed a moderate reduction of 9.1% and 9.9% compared to the control mix, respectively. However, increasing the replacement ratio to 75% and 100% resulted in greater reductions of 21.1% and 24.7% compared to the control mix, respectively. For the 28-day results, compressive strength reductions of 15.9%, 17.3%, and 18.3% were observed for mixes with 25%, 75%, and 100% wash water replacement compared to the control mix, respectively. Notably, the mix with 50% wash water replacement exhibited only an insignificant reduction compared to the control sample. This minor variation could be attributed to the natural variability in concrete properties, which can account for fluctuations of up to 15%. A comparison of compressive strength results at 7 and 28 days for all mixes is illustrated in Fig. 5.

**Table 3:** Concrete compressive strength test results according to BS EN 12,390^17^.

Concrete sample	7-day compressive strength (MPa)		28-day compressive strength (MPa)	
	*f*_cu exp_	% *f*_cu_*	*f*_cu exp_	% *f*_cu_*
C	16.6 ± 1.1	–	25.6 ± 1.1	–
M25	15.1 ± 0.25	90.9%	21.5 ± 0.55	84.1%
M50	14.9 ± 0.75	90.1%	25.5 ± 0.55	99.9%
M75	13.1 ± 0.7	78.9%	21.1 ± 1.13	82.7%
M100	12.5 ± 0.90	75.3%	20.9 ± 0.15	81.9%

*Percentage of compressive strength of the samples incorporated wash water (*f*_cu exp_) compared to the control sample (C).

It is worth mentioning that ECP 203^21^ permits a maximum reduction of 10% in compressive strength when using non-potable water. Based on this criterion, the mixes with 25% and 50% wash water replacement satisfied the code requirement at 7 days, which aligns with the findings reported by Klus et al.^6^. Conversely, the mixes of 75% and 100% replacement ratios exceeded this permissible limit.

Although the total mixing water content was maintained constant for all mixes, the presence of residual cement particles, fines, and other impurities in wash water can reduce the amount of free water available for cement hydration. This reduction in free water influences hydration kinetics and can weaken the bonding within the cementitious matrix. For mixes with higher replacement levels (M75 and M100), the observed reduction in compressive strength can be attributed to the combined effects of reduced effective water availability and disturbed hydration mechanisms. The presence of dissolved salts and fine residues can interfere with normal hydration processes by disrupting the formation and continuity of calcium–silicate–hydrate (C–S–H) gel.

At lower wash water replacement ratios (M25 and M50), the presence of fine particles can produce a beneficial micro-filler effect, improving particle packing density and partially compensating for compressive strength reduction. However, at higher replacement ratios, excessive fines and impurities can hinder the hydration process and weaken the interfacial transition zone between the cement paste and aggregates, ultimately resulting in reduced compressive strength. These results are consistent with the findings of Aruntaş^13^, they reported that wash water replacement has a negative impact on the compressive strength of concrete, particularly at higher replacement ratios.

The relationship between the ratio of the compressive strength of specimens produced with wash water to that of the control specimen produced with potable water (*f*_cw_/*f*_cp_) and the percentage of wash water replacement (*WWr*) is illustrated in.

**Fig. 6:**
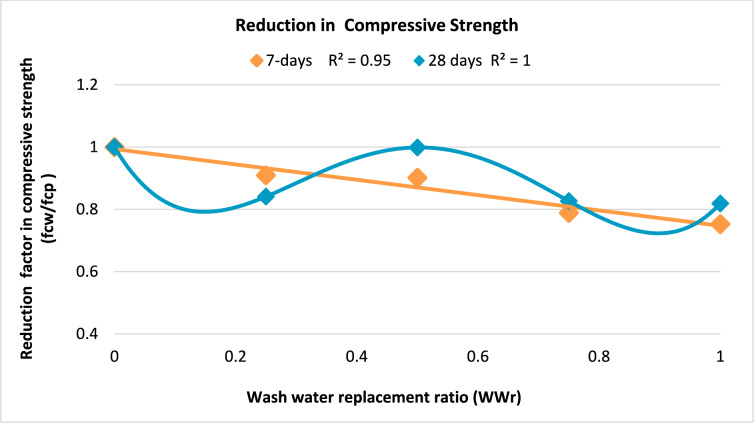
Relation between reduction in compressive strength and wash water replacement ratio at ages of 7 and 28 days.

Figure 6 for both 7 and 28 days. Here, *f*_cw_ represents the compressive strength of the concrete produced with wash water, while *f*_cp_ denotes the compressive strength of the control mix made with potable water. The parametric study demonstrated the negative impact of the wash water replacement ratio on the compressive strength of concrete.

Based on the regression analysis of the experimental data, empirical formulas were developed to predict the reduction factor (*f*_cw_/*f*_cp_) in concrete compressive strength as a function of the wash water replacement ratio (*WWr*). The proposed Eqs. (2) and (3), which estimate the reduction in compressive strength at 7 and 28 days, respectively, are expressed as follows:

2 $${f}_{\mathrm{cw}}/{f}_{\text{cp }}\left(7\text{ days}\right)=- 0.2463 (WW\mathrm{r}) + 0.9936$$

3 $${f}_{\mathrm{cw}}/{f}_{\text{cp }}\left(28\text{ days}\right)=12.169{\left(WW\mathrm{r}\right)}^{4}-25.151{\left(WW\mathrm{r}\right)}^{3}+16.074{\left(WW\mathrm{r}\right)}^{2}-3.2729\left(WWr\right)+1$$

where: *f*_cw_ is the compressive strength of the concrete produced using wash water from the ready-mix plant. *f*_cp_ is the compressive strength of the concrete produced using potable water (control mix). *WWr* is the wash water replacement ratio in the concrete mix.

To validate the proposed equations, Table 4 compares the predicted compressive strength values (*f*_cu pre_) obtained from the proposed equations with the corresponding experimental results (*f*_cu exp_) at 7 and 28 days. The predicted values were calculated for mixes incorporating 25%, 50%, 75%, and 100% wash water replacement, based on the average compressive strength of the control mix (with 0% wash water replacement). The control mix achieved compressive strengths *f*_cp_ of 16.6 MPa and 25.5 MPa at 7 and 28 days, respectively.

**Table 4:** Validation of proposed equations.

Concrete Sample	7-day compressive strength (MPa)			28-day compressive strength (MPa)		
	Experimental (*f*_cu exp_)	Predicted (*f*_cu pre_)	(*f*_cu exp_/*f*_cu pre)_*	Experimental (*f*_cu exp_)	Predicted *(f*_cu pre_)	(*f*_cu exp_/*f*_cu pre)_*
M25	15.3	15.5	0.99	21	22.45	0.94
	14.8		0.96	21.4		0.95
	15.1		0.98	22.1		0.98
M50	14.2	16.5	0.86	25.5	25.05	1.01
	14.9		0.90	26.1		1.01
	15.7		0.95	25.0		1.01
M75	13.7	13.4	1.02	20.8	20.54	1.01
	13.2		0.98	20.2		0.98
	12.3		0.92	22.4		1.09
M100	13	12.4	1.05	21.1	21.23	0.99
	11.4		0.92	20.9		0.98
	13		1.05	20.8		0.98

*Ratio of the experimental result (*f*_cu exp_) to the predicted value (*f*_cu pre_) of concrete compressive strength.

**Fig. 7:**
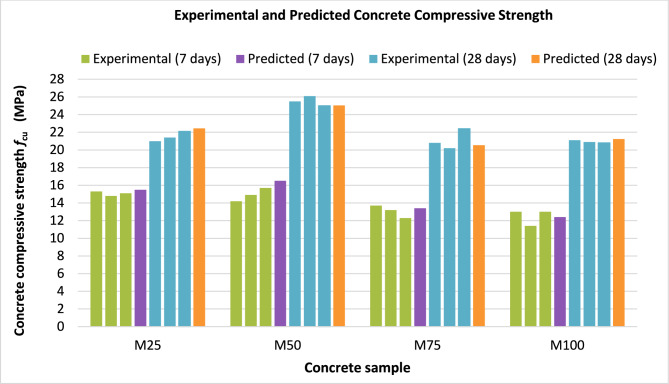
Experimental and predicted concrete compressive strength for concrete samples at ages of 7 and 28 days.

In the present study, the proposed empirical equations were developed and validated within the scope of the experimental programme to provide preliminary, performance-based indicators for estimating compressive strength reduction in concrete incorporating wash water within the investigated replacement ratios. Figure 7 illustrates the relationship between the experimental and predicted compressive strength values for concrete samples with different water replacement ratios at ages of 7 and 28 days. The results demonstrate that the proposed equations show good agreement with the experimental data, indicating that it can be effectively used to estimate the reduction in compressive strength due to wash water replacement within the studied range. In addition, the uncertainty in compressive strength was calculated using Type A uncertainty, as expressed in Eq. (4).

4 $${U}_{A}=\frac{standard\,deviation}{\sqrt{n}}$$

where: *U*_*A*_ is the uncertainty level in concrete compressive strength. n is the number of specimens.

The resulting uncertainty was then multiplied by 2 to achieve a 95% confidence level. The maximum calculated uncertainty was 1.3% for the compressive strength of the concrete cubes, indicating high reliability and consistency of the experimental results.

### Splitting tensile test results

**Fig. 8:**
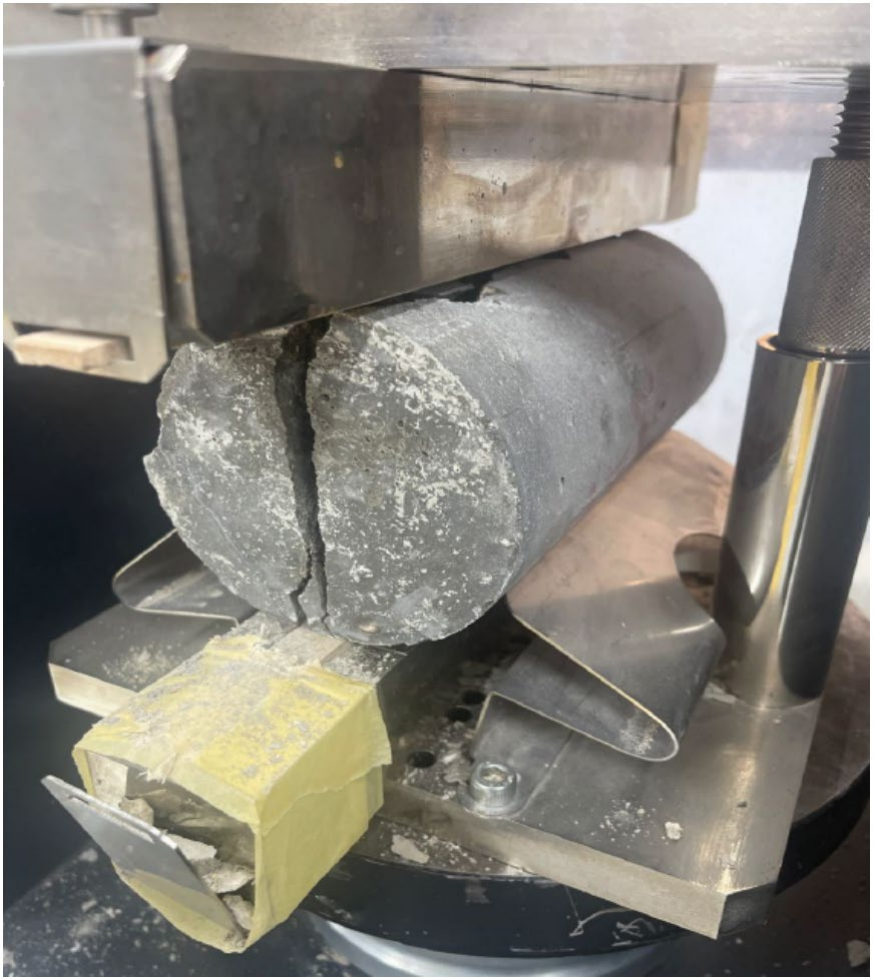
Failure mode observed during the splitting tensile strength test.

**Fig. 9:**
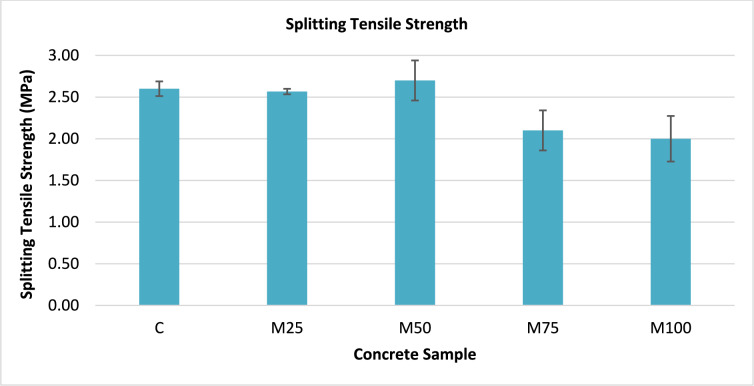
Splitting tensile strength of the concrete mixes.

The splitting tensile strength test was conducted on three cylindrical specimens, each measuring 150 × 300 mm, to evaluate the tensile behaviour of the concrete mixes. The typical failure mode observed during the test is illustrated in Fig. 8. The corresponding test results are summarized in Table 5. The uncertainty for the test was calculated using Eq. (4), and the resulting uncertainty was 0.54%, indicating high consistency in the measurements. The analysis of the results showed a clear trend with the increasing proportion of wash water in the concrete mix, as summarized in Table 5. The control mix (C), prepared using 100% potable water, achieved a splitting tensile strength (${f}_{t}$) of 2.60 MPa, serving as the reference value. For the mixes containing 25% and 50% wash water replacement, the splitting tensile strengths were 2.57 MPa and 2.70 MPa, corresponding to approximately 99% and 104% of the control mix strength, respectively. These variations fall within the normal range of experimental variability for concrete mixes, indicating that moderate levels of wash water replacement, up to 50% replacement, do not significantly affect the splitting tensile strength. A comparison of the splitting strength results for all mixes is illustrated in Fig. 9.

**Table 5:** Splitting tensile test results.

Concrete sample	Splitting tensile strength *f*_t_ (MPa)	
	Average	% *f*_t_*
C	2.60 ± 0.15	–
M25	2.57 ± 0.05	99%
M50	2.70 ± 0.41	104%
M75	2.10 ± 0.41	81%
M100	2.00 ± 0.47	77%

*Percentage of the average tensile strength for the sample incorporating wash water compared to the tensile strength of the control sample.

However, as the wash water replacement increased to 75% and 100%, the tensile strength showed a noticeable decline, recording values of 2.10 MPa and 2.00 MPa, respectively. These correspond to 81% and 77% compared to the strength control sample. This reduction in tensile strength at higher replacement levels can be attributed to the excessive presence of contaminants, unreacted cement particles, and fine solids in the wash water. These impurities can interfere with the cement hydration process, weaken the bond between the cement paste and aggregates, and increase the formation of voids and microcracks within the concrete matrix. Consequently, the concrete tensile strength is decreased due to the formation of weaker interfacial transition zones and greater susceptibility to cracking under tensile stress.

### Electrical resistivity test

The electrical resistivity test conducted in this study evaluates the long-term durability and corrosion resistance of concrete mixes incorporating wash water. Unlike conventional durability tests, such as chloride penetration or half-cell potential methods, the electrical resistivity test is a rapid, non-destructive, and portable technique, requiring approximately one minute per measurement. This makes it suitable for both laboratory and on-site evaluation of concrete quality and corrosion potential.

The test was performed on saturated surface dry (SSD) specimens at 28 days of curing, and the corresponding results are presented in Table 6. A positive correlation was observed between the ratios of wash water used in the mixes and the measured electrical resistivity values compared to the control sample. Specifically, as the wash water replacement ratio increased, both the potential difference (ΔV) and the electrical resistivity (*ρ*) of the concrete increased. The results indicate that electrical resistivity increased by approximately 44% and 60% for the mixes incorporating 25% and 50% wash water replacement, respectively, relative to the control mix. Furthermore, increasing the wash water replacement ratio to 75% and 100% led to resistivity values that are double and six times higher, respectively, compared to the control sample.

**Table 6:** Results of the electrical resistivity test.

Concrete sample	Potential difference (ΔV) (mV)	Electrical resistivity (*ρ*) kΩ.cm
C	5	25
M25	6	36
M50	8	40
M75	15.5	78.5
M100	35	178

Although the wash water contains dissolved salts, the observed increase in electrical resistivity at higher replacement ratios can be attributed to pore structure refinement dominating over ionic conductivity effects. The presence of ultra-fine cementitious particles and hydration by-products in the treated wash water contributes to a micro-filler effect, partially blocking capillary pores and increasing pore tortuosity. This reduction in pore connectivity limits ionic mobility within the concrete matrix, resulting in higher electrical resistivity. Similar behaviour has been reported by Yao et al.^3^ and Sandrolini et al.^22^, who observed increased durability due to pore-filling effects of fine solids.

The increase in electrical resistivity with higher wash water content is favorable for corrosion resistance, particularly under saturated or aggressive exposure conditions. However, this improvement in durability coincides with a reduction in compressive strength, as discussed in Sect. 3.3. This can be explained by the dual effect of the salts and suspended solids present in the wash water. While fine particles may refine the pore structure and increase resistivity, excessive solids and chemical residues can disrupt normal cement hydration and weaken the interfacial transition zone, ultimately leading to reduced compressive strength. These findings are consistent with Gupta et al.^23^. They reported an increase in concrete density when using grey wastewater, attributed to the deposition of fine solids that partially fill pores and reduce permeability.

Furthermore, the treated wash water exhibited an elevated alkalinity (pH = 9.1), which contributes to maintaining a passive environment for embedded steel reinforcement, thereby reducing corrosion risk. Additionally, the chloride and sulfate concentrations were significantly below the threshold limits specified by ASTM C1602 and BS EN 1008, indicating a low likelihood of chloride-induced corrosion or sulfate attack. Therefore, the increase in electrical resistivity observed in this study is consistent with improved corrosion resistance under the investigated conditions.

## Conclusions

In this study, an experimental programme was conducted to evaluate the effect of using wash water from ready-mix concrete plants as a sustainable alternative for the partial and full replacement of potable water in concrete production. The experimental programme included concrete mixes incorporating 25%, 50%, 75%, and 100% wash water replacement ratios. The influence of wash water on the fresh properties, mechanical performance, and durability of concrete was systematically investigated. Based on the results and analysis presented in this study, the following conclusions can be drawn:

- The treated wash water satisfied the chemical requirements of ASTM C1602, BS EN 1008, and ECP 203 for concrete mixing water. Its higher can enhance the corrosion resistance of steel reinforcement, provided that non-reactive aggregates are used to mitigate potential alkali–silica reaction risks.
- The incorporation of wash water resulted in a reduction in workability, with slump values decreasing by up to 50%. This reduction is attributed to fine suspended cementitious particles and dissolved solids, which increase the surface area of the mix and consequently the water demand. This reduction in workability can be effectively mitigated using suitable chemical admixtures.
- Compressive strength decreased with increasing wash water replacement ratio, with a maximum reduction of 18.3% at 28 days. However, concrete mixes incorporating up to 50% treated wash water met the strength requirements of BS EN 12,390 and ECP 203, indicating that 50% replacement can be considered a sustainable and safe alternative without compromising mechanical performance.
- Concrete mixes incorporating up to 50% wash water exhibited splitting tensile strengths comparable to the control mix, indicating no adverse effect on tensile strength. Higher replacement ratios of 75% and 100% resulted in a maximum tensile strength reduction of 23% compared to the control sample.
- Electrical resistivity increased consistently with wash water replacement, rising by 44% and 60% at 25% and 50% replacement levels, and by approximately two and six times at 75% and 100% replacement, respectively. This increase is attributed to the micro-filler effect of ultra fine cementitious particles in the treated wash water, which reduces pore connectivity and increases pore tortuosity. The observed trend indicates a potential improvement in corrosion resistance due to the refinement of the concrete pore structure.
- Empirical equations were developed and validated within the scope of this study to estimate compressive strength reduction as a function of wash water replacement ratio. These equations provide preliminary performance-based guidance for mix design but are limited to the material and replacement ranges investigated.
- Based on the experimental results, replacing up to 50% of potable mixing water with treated wash water can be implemented in ready-mix concrete plants without significant loss of mechanical performance while offering durability benefits. Practical implementation may be achieved through simple and cost-effective treatment measures such as sedimentation and basic filtration, combined with controlled blending and routine monitoring to ensure compliance with ASTM C1602 and BS EN 1008. This approach can reduce freshwater consumption, lower wastewater disposal costs, and support sustainability objectives without major modifications to existing batching infrastructure.

### Limitations and recommendations for future work

- This study was limited to treated wash water obtained from a single ready-mix concrete plant; therefore, the results are applicable only within the investigated materials and operating conditions. Since wash water characteristics may vary between plants, future studies should consider multiple sources to improve the general applicability of the findings
- Durability assessment was limited to electrical resistivity as a rapid, non-destructive indicator. Future research should include complementary durability indicators such as chloride migration and water permeability to enable a more comprehensive evaluation of long-term performance. Future studies should incorporate detailed physical characterization of wash water, including particle size distribution and turbidity, as well as advanced microstructural techniques such as SEM and XRD, to better elucidate hydration mechanisms and pore structure refinement.
- The proposed empirical equations for compressive strength prediction were developed within the scope of the present experimental dataset and are valid only for the investigated wash water replacement ratios. Further experimental studies involving different concrete compositions, wash water sources, and curing conditions are required to expand the dataset and support the development of generalized predictive models suitable for standardization.

## Data Availability

The datasets used and/or analyzed during the current study are available from the corresponding author on reasonable request.
